# A differential role for CXCR4 in the regulation of normal versus malignant breast stem cell activity

**DOI:** 10.18632/oncotarget.1169

**Published:** 2013-07-30

**Authors:** Matthew P. Ablett, Ciara S. O'Brien, Andrew H. Sims, Gillian Farnie, Robert B. Clarke

**Affiliations:** ^1^ Breast Biology Group, Institute of Cancer Sciences, Paterson Building, University of Manchester, Wilmslow Road, Manchester,M20 4BX,UK; ^2^ Cancer Stem Cell Research, Institute of Cancer Sciences, Paterson Building, University of Manchester, Wilmslow Road, Manchester,M20 4BX,UK; ^3^ Applied Bioinformatics of Cancer, Edinburgh Cancer Research Centre, Institute of Genetics and Molecular Medicine, Crewe Road, Edinburgh, EH4 2XU, UK

**Keywords:** CXCR4, stem cells, breast cancer, mammospheres, AMD3100, SDF-1

## Abstract

C-X-C chemokine receptor type 4 (CXCR4) is known to regulate lung, pancreatic and prostate cancer stem cells. In breast cancer, CXCR4 signalling has been reported to be a mediator of metastasis, and is linked to poor prognosis. However its role in normal and malignant breast stem cell function has not been investigated.

Anoikis resistant (AR) cells were collected from immortalised (MCF10A, 226L) and malignant (MCF7, T47D, SKBR3) breast cell lines and assessed for stem cell enrichment versus unsorted cells. AR cells had significantly higher mammosphere forming efficiency (MFE) than unsorted cells. The AR normal cells demonstrated increased formation of 3D structures in Matrigel compared to unsorted cells. *In vivo*, SKBR3 and T47D AR cells had 7- and 130-fold enrichments for tumour formation respectively, compared with unsorted cells.

AR cells contained significantly elevated CXCR4 transcript and protein levels compared to unsorted cells. Importantly, CXCR4 mRNA was higher in stem cell-enriched CD44^+^ /CD24^−^ - patient-derived breast cancer cells compared to non-enriched cells. CXCR4 stimulation by its ligand SDF-1 reduced MFE of the normal breast cells lines but increased the MFE in T47D and patient-derived breast cancer cells. CXCR4 inhibition by AMD3100 increased stem cell activity but reduced the self-renewal capacity of the malignant breast cell line T47D. CXCR4 + FACS sorted MCF7 cells demonstrated a significantly increased MFE compared with CXCR4- cells. This significant increase in MFE was further demonstrated in CXCR4 over-expressing MCF7 cells which also had an increase in self-renewal compared to parental cells. A greater reduction in self-renewal following CXCR4 inhibition in the CXCR4 over-expressing cells compared with parental cells was also observed.

Our data establish for the first time that CXCR4 signalling has contrasting effects on normal and malignant breast stem cell activity. Here, we demonstrate that CXCR4 signalling specifically regulates breast cancer stem cell activities and may therefore be important in tumour formation at the sites of metastases.

## INTRODUCTION

The requirement of the breast to undergo the high level of proliferation observed during puberty and each round of pregnancy suggests the presence of a stem cell population within the breast [1]. Unequivocal evidence for the presence of stem cells in the murine mammary gland has been reported from a variety of studies [2-4]. There is evidence that a population of cells within breast cancer display stem cell properties Al-Hajj *et al.* [5]. In a clinical study, Li *et al.* [6] demonstrated enrichment of the breast cancer stem cell population (identified by flow cytometry and mammosphere assay *in vitro*) in response to chemotherapy. Many groups have enriched for functional breast cancer stem cells using ESA^+^/CD44^+^/CD24^−/low^ [7-10]. In addition, mammosphere culture has been used to assess both normal and cancer stem cell activity. Recent evidence suggests that the mammosphere assay acts an accurate *in vitro* tumour model, particularly of aggressive tumours [11, 12]. Dontu *et al.* [13] utilised this assay to enrich for breast stem cells by harvesting mammospheres formed from cells which survived non-adherent culture conditions, the anoikis-resistant cells. Harrison *et al.* [10] reported that anoikis resistant cells had increased CD44^+^/CD24^−^ expression and could be used to enrich for breast cancer stem cells.

CXCR4 is the most common chemokine receptor expressed on tumour cells and has been detected in 23 different types of cancer [14, 15]. CXCR4 signalling has been linked with aggressiveness and the promotion of metastasis, with cells expressing the receptor homing to tissues secreting SDF-1 (stromal cell-derived factor-1). Muller *et al.* [16] and others have demonstrated that CXCR4/SDF-1 signalling *in vivo* regulates breast cancer metastases [17-22]. CXCR4 expression has been detected in the stem cell population of lung, pancreatic and prostate tumours [23-25].

In the current study we used anoikis-resistance to enrich for normal and malignant breast stem cells. Stem cell enrichment was validated both *in vitro* and *in vivo*. CXCR4 expression was found to be up-regulated in the stem cell-enriched populations and we show that CXCR4 signalling has contrasting effects on normal and malignant breast stem cell activity. Signalling through CXCR4 is found to specifically regulate self-renewal of malignant stem cells which may account for its role in breast cancer progression and metastasis.

## RESULTS

### Anoikis resistance enriches for mammosphere-initiating, normal colony-forming and tumour-initiating cells

**Figure 1:**
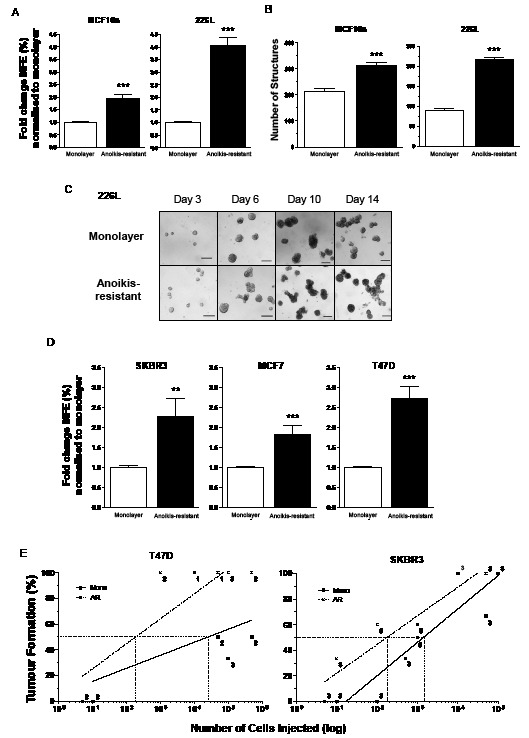
Normal and malignant stem cells are anoikis-resistant Anoikis-resistant (AR) cells were harvested from 2 normal (MCF10a and 226L) and 3 malignant (SKBR3, MCF7 and T47D) breast cell lines and demonstrated stem cell enrichment. AR cells from MCF10a and 226L cell lines formed more mammospheres (A) and 3D structures in Matrigel (B and C) compared with unsorted monolayer cells. AR cells from SKBR3, T47D and MCF7 cell lines formed more mammospheres (D) and formed tumours *in vivo* more efficiently than their monolayer cells (E – SKBR3 and T47D only). Mammosphere results represent 3 independent experiments in triplicate (A and D). Matrigel results represent 2 independent experiments in quadruplicate (B). The numbers beside each data point represent the number of mice tested with each amount of cells (E). MFE – mammosphere forming efficiency, Mono – monolayer cells, AR – 12 hour anoikis-resistant cells. Scale bar (C) 100μm, error bars ± S.E.M., ** p<0.01, *** p<0.001.

Anoikis-resistant cells harvested after 12 hours in mammosphere culture from two normal breast cell lines demonstrated significant increases in mammosphere forming potential compared with unsorted monolayer cells (Figure 1A – 226L: 4.0-fold increase, p<0.001; MCF10a: 1.9-fold increase, p<0.001). The degree of stem cell enrichment was also assessed *in vitro* using 3D-Matrigel culture (Figures 1B and C). The anoikis-resistant cells formed significantly more structures than unsorted monolayer cells for both cell lines (226L: 2.4 fold increase, MCF10a: 1.5 fold increase, both p<0.001). This significant increase in structures formed was seen across all sizes except those greater than 300 μm where the number of structures formed was too low to gain significance (data not shown).

Twelve hour anoikis-resistant cells from the 3 malignant cell lines, MCF7, SKBR3 and T47D also demonstrated a significant increase in the number of mammospheres formed compared with monolayer cells (Figure 1D – MCF7: 1.8-fold increase, p<0.001; T47D: 2.7-fold increase, p<0.001; SKBR3: 2.3-fold increase, p<0.01). Previous work by Harrison *et al.* [10] demonstrated that the anoikis-resistant population of MCF7 cells is enriched for stem cells both *in vitro* and *in vivo* (5.7-fold and 12-fold respectively). However, until now, no studies have demonstrated the tumorigenic potential of the anoikis-resistant population of T47D or SKBR3 cell lines *in vivo*. We therefore injected limiting dilutions of T47D or SKBR3 cells into immunocompromised mice to investigate the tumour initiating capacity of the anoikis-resistant cells versus monolayer cells. It can clearly be seen that anoikis-resistant cells form tumours more efficiently than monolayer cells (Figure 1E – fold change at 50% tumour formation, T47D: 130-fold increase and SKBR3: 7-fold increase).

### Analysis of gene expression in anoikis-resistant cells reveals up-regulation of CXCR4 in normal and malignant breast cell lines

Changes in gene expression between the populations of anoikis-resistant cells (collected after 8 or 12 hours in mammosphere culture) and monolayer cells for the five cell lines were analysed using an Agilent custom microarray. We identified genes which were differentially expressed between monolayer and the 2 anoikis-resistant cell populations. Initial analysis revealed no significant differences between the 8 and 12 hour anoikis-resistant cells in both the normal and malignant cell lines. However, there were more genes differentially expressed between 12 hour anoikis-resistant cells (compared to monolayer) than between 8 hour anoikis-resistant cells (compared to monolayer) supporting the hypothesis that greater stem cell-enrichment is achieved after 12 hours in mammosphere culture (data not shown). Fewer genes were differentially expressed between the anoikis-resistant and monolayer populations of the normal cell lines compared with the malignant cell lines.

**Figure 2:**
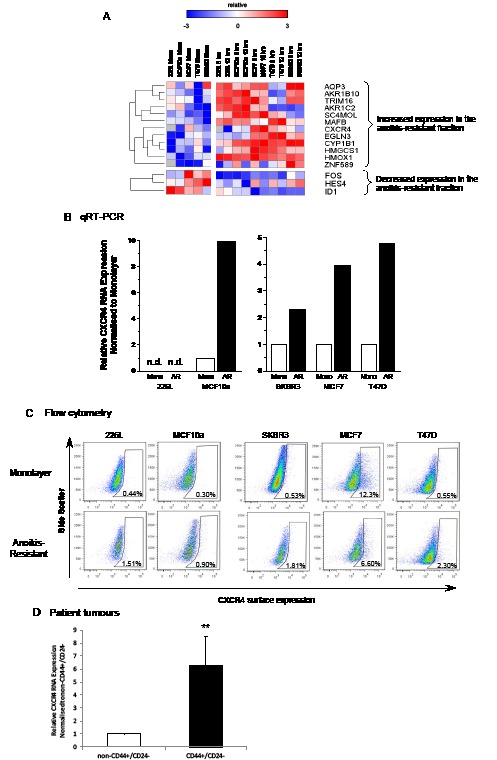
CXCR4 RNA and protein expression is increased in anoikis-resistant cells Gene expression of the anoikis-resistant fraction versus monolayer cells of 2 normal (MCF10a and 226L) and 3 malignant (SKBR3, MCF7 and T47D) breast cell lines were analysed using an Agilent custom microarray. 12 genes increased >2-fold expression in the anoikis-resistant population versus monolayer cells while 3 genes significantly decreased >2-fold expression averaging expression across all cell lines (A). Quantitative RT-PCR and FACS confirmed increased gene and cell surface expression of CXCR4 in 4 out of the 5 cell lines analysed compared with monolayer cells (B and C). CXCR4 transcript levels were also found to be significantly higher in CD44+/CD24− flow sorted cells from Creighton et al. 2009 [28] (D). Pearson correlation was used for hierarchical clustering of gene expression (rows), red = high and blue = low relative to mean (white), grey = no expression. Mono – monolayer cells, AR – 12 hour anoikis-resistant cells, n.d. – not detectable. FACS plots representative of 3 independent experiments, (D) n = 14, error bars ± S.E.M., ** p<0.01.

Figure 2A shows genes which had a greater than 2-fold difference in expression between monolayer and anoikis-resistant populations. Several of these genes have previously been associated with stem cell functions: MAFB and ZNF589 have been shown to be important in lineage-specific haematopoiesis [26, 27], while HES4 is a down-stream target of the Notch signalling pathway which has been shown to affect breast cancer stem cell activity [10]. CXCR4 has previously been shown to be important in metastasis of breast cancer and is highly expressed in the cancer stem cell population of other epithelial cancers [23-25]. We therefore chose to focus our attention on the expression and role of CXCR4 signalling in normal and malignant breast stem cells.

The gene expression levels of CXCR4 in the 12 hour anoikis-resistant and monolayer cells were validated by qPCR, while flow cytometry was used to assess CXCR4 cell surface expression. An increase in CXCR4 transcripts were observed in the anoikis-resistant cells compared with the monolayer cells for 4 out of the 5 breast cell lines (Figure 2B). CXCR4 expression was too low to be detected in either population of 226L cells. Both normal cell lines showed low levels of cell surface expression of CXCR4 in monolayer (226L – 0.44%, MCF10a – 0.30%). The expression of CXCR4 remained low, but significantly increased in the anoikis-resistant cell population for both cell lines (226L – 3.6-fold increase, n=3, p<0.001; MCF10a – 3.0-fold increase, n=3, p<0.05). Cell surface expression of CXCR4 was significantly increased in the anoikis-resistant cell populations of T47D (5.3-fold change, n=3, p<0.05) and SKBR3 (2.9-fold change, n=3, p<0.05). However, there was a significant decrease in cell surface expression of CXCR4 in the anoikis-resistant cell population of the malignant cell line which expressed the most CXCR4 in monolayer, MCF7 cells (12.1% in monolayer, 0.5-fold change in anoikis-resistant population, n=3, p<0.05). MCF7 anoikis-resistant cells were the only cell line to decrease in cell surface expression (Figure 2C). To obtain supporting data from patient tumours, we obtained CXCR4 transcript levels from Creighton et al. [28] where breast cancer stem cell-enriched populations (CD44^+^/CD24^−^) were sorted by flow cytometry and gene expression was analysed (Figure 2D). The data show an average 6-fold higher CXCR4 expression in patient-derived breast cancer stem cells (n=14, p<0.01).

### Effects of stimulation and inhibition of CXCR4 on mammosphere-formation/self-renewal of cell lines and patient-derived tumours

**Figure 3:**
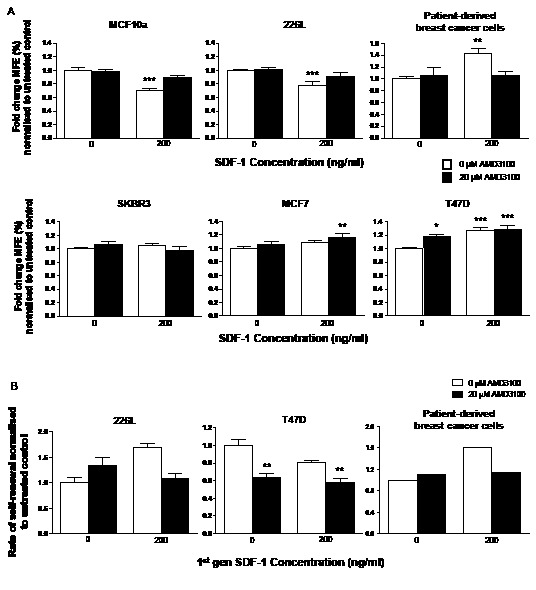
Inhibition and stimulation of CXCR4 signalling has differing effects on stem cell activity of normal and malignant breast cell lines and patient-derived cells SDF-1 and AMD3100 was used to stimulate and inhibit CXCR4 signalling respectively in the mammosphere assay. Stimulation of CXCR4 signalling reduced the number of mammospheres formed in the normal breast cell lines MCF10a and 226L while it increased mammosphere formation in the malignant cell line T47D and cells derived from patients with breast cancer (A). Combination treatment with SDF-1 and AMD3100 increased mammosphere formation in both MCF7 and T47D cell lines but had no effect on SKBR3 cells. Treatment with AMD3100 reduced stem cell self-renewal of T47D cells (B). SDF-1 treatment increased self-renewal of the single patient derived sample tested for 2^nd^ generation mammosphere formation. Both treatments had no significant effects on self-renewal of 226L cells. 1^st^ generation mammosphere results (A) represent 3 independent results in triplicate. 226L and T47D 2^nd^ generation mammosphere results represent 3 independent results. Patient-derived cells were obtained from 2 ascites samples and primary established cell line (from an ascites). All statistics are comparing mammosphere formation against untreated controls (far left bar of each chart). MFE – mammosphere forming efficiency, error bars ± S.E.M., * p<0.05, ** p<0.01, *** p<0.001.

To examine the effects of CXCR4 signalling on stem cell activity, the CXCR4 ligand, SDF-1 and the CXCR4 antagonist, AMD3100 were used to stimulate or inhibit signalling respectively, either alone, or in combination (Figure 3). Mammosphere forming efficiency (MFE) was significantly reduced with the addition of SDF-1 (p<0.001) whereas AMD3100 had no effect on either of the immortalised normal breast cell lines. However, addition of the antagonist in combination with SDF-1 rescued the reduction seen with SDF-1 treatment alone and increased the MFE of both cell lines back to control levels (Figure 3A, p<0.001 for both).

Two human ascites samples taken from patients with breast cancer were also treated with AMD3100 and SDF-1, along with a cell line originally established from a primary ascites sample. SDF-1 treatment alone significantly increased MFE (p<0.01) while AMD3100 alone had no effect (Figure 3A). Combination treatment reduced MFE back to the level of untreated samples (p<0.01).

Single and combination treatment of AMD3100 and SDF-1 in mammosphere culture was repeated with the 3 malignant cell lines MCF7, T47D and SKBR3 (Figure 3A). Only the MFE of the T47D cell line was significantly increased in response to AMD3100 (p<0.05). SDF-1 treatment alone increased MFE in both MCF7 and T47D cell lines, but again, significance was only reached in the T47D cell line (p<0.001). Interestingly, combination treatment of AMD3100 and SDF-1 to MCF7 and T47D cells in mammosphere culture significantly increased MFE further compared with no treatment (MCF7 – p<0.01, T47D – p<0.001). AMD3100 or SDF-1 alone or combination had no effect on the MFE of the ER negative cell line, SKBR3. Differences in the response of patient-derived samples to SDF-1 compared to the cell lines is likely due to high endogenous cytokine levels in the fluid (Mean 5413.3pg/ml) from which the cells were obtained. In contrast, there was 7-fold less SDF-1 present in T47D cell line-conditioned medium and several hundred-fold lower levels in MCF-7 and SKBR3 cells (Table 1). Thus, control MFE may represent an SDF-1 withdrawal response while treatment may represent maintenance of cytokine exposure.

**Table 1:** An ELISA was used to measure SDF-1 concentrations in the metastatic fluid collected from human patient derived ascites samples and from the media of cell lines grown in culture for 3 days Fold change for each cell line was calculated versus the mean SDF-1 concentration from the 3 ascites samples. N.d. – not detected, means calculated from 3 individual samples in duplicate.

Fold change vs. ascites	Sample	Mean SDF-1 concentration (pg/ml)
	Human patient-derived ascites	5413.3
x 7	T47D	767.5
x 159	MCF7	34.0
x 381	SKBR3	14.2
x 588	226L	9.2
x ∞	MCF10a	n.d.

First generation mammospheres from 226L, T47D cells and one of the ascites samples were dissociated and re-plated into a mammosphere assay in the absence of secondary treatment to assess the effects of first generation treatment on stem cell self-renewal (Figure 3B). A small increase in self-renewal was seen with single treatments of SDF-1 or AMD3100 for the 226L cell line while T47D cells had a marked decrease in self-renewal of mammospheres treated with AMD3100 (both alone and in combination with SDF-1) during second generation culture (p<0.01), while SDF-1 had no effect on self-renewal. For the ascites sample, first generation cells treated with SDF-1 alone showed an increase in self-renewal. This was reduced when AMD3100 was added in combination with SDF-1, while AMD3100 treatment alone had no effect.

### Mammosphere formation/self-renewal in FACS-sorted CXCR4-positive and negative populations treated with SDF-1 and AMD3100

**Figure 4:**
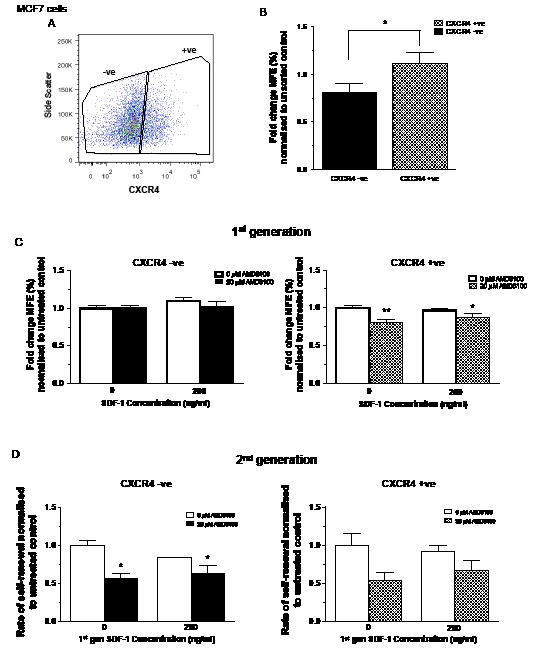
Effects of inhibition and stimulation of CXCR4 signalling on stem cell activity of CXCR4 sorted MCF7 cells MCF7 cells were sorted for cell surface expression of CXCR4 (A). CXCR4 positive cells demonstrated increased mammosphere formation compared with CXCR4 negative cells (B). Inhibition of CXCR4 signalling using AMD3100 reduced stem cell activity of CXCR4 positive cells while it had little effect on 1^st^ generation CXCR4 negative cells (C). CXCR4 positive and negative cells showed similar results for 2^nd^ generation mammosphere formation (D). No further treatment was added to 2^nd^ generation mammosphere cultures. 1^st^ generation mammosphere results (B and C) represent 3 independent results in triplicate. 2^nd^ generation mammosphere results represent 3 independent results. All statistics are comparing mammosphere formation against untreated controls (far left bar of each chart). MFE – mammosphere forming efficiency, error bars ± 1 S.E.M., * p<0.05, ** p<0.01.

In an attempt to further resolve the effects of CXCR4 on stem cell activity, cells positive for surface expression of CXCR4 were sorted using FACS. The MCF7 cell line was chosen due to its high CXCR4 expression. Both positive and negative fractions were collected and purity checks performed (Figures 4a and data not shown).

CXCR4 positive FACS cells had higher MFE than CXCR4 negative cells (p < 0.05, Figure 4B). The CXCR4 negative cells showed little change in MFE in response to SDF-1 or AMD3100 treatment (Figure 4C) while the first generation MFE of the CXCR4 positive fraction of MCF7 cells mimicked the response of secondary generation unsorted T47D cells, with AMD3100 significantly reducing the MFE independent of SDF-1 treatment (AMD3100 alone; p<0.01, combination; p<0.05). Second generation data for the negative CXCR4 fraction demonstrated a significant decrease in MFE in response to AMD3100 primary treatment alone and in combination with SDF-1 (Figure 4D). A decrease was seen for the same treatment conditions in the positive CXCR4 fraction however this did not reach significance. Neither population showed any effect of primary SDF-1 treatment on second generation MFE.

### CXCR4 over-expression induces mammosphere formation/self-renewal and is inhibited by AMD3100

**Figure 5:**
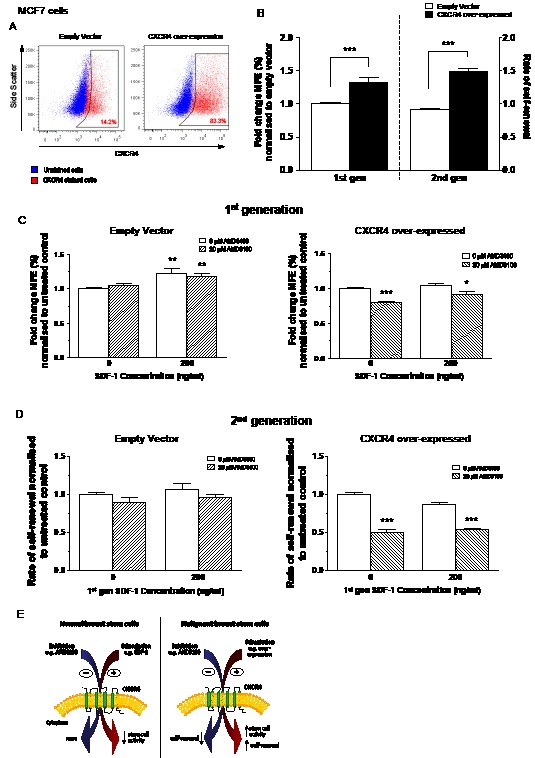
Effects of inhibition and stimulation of CXCR4 signalling on stem cell activity of CXCR4 over-expressing MCF7 cells Cell surface expression of CXCR4 increased 5.9-fold after retroviral infection with human codon optimised CXCR4 cDNA (A). Over-expression of CXCR4 increased both stem cell activity and self-renewal of MCF7 cells in the mammosphere assay (B). MCF7 cells over-expressing CXCR4 showed sensitisation to the effects of CXCR4 inhibition by AMD3100 causing both a reduction in stem cell activity (C) and self-renewal (D). A proposed model for CXCR4 signalling in normal and malignant breast stem cells is shown in (E); Stimulation of CXCR4 signalling decreases stem cell activity in normal breast stem cells. The opposite effect occurs in malignant breast stem cells while also increasing stem cell self-renewal. Inhibition of CXCR4 signalling has no measurable effect on normal stem cells but reduces malignant stem cell self-renewal. No further treatment was added to 2^nd^ generation mammosphere cultures. 1^st^ generation mammosphere results (B and C) represent 3 independent results in triplicate. 2^nd^ generation mammosphere results represent 3 independent results. All statistics are comparing mammosphere formation against untreated controls. MFE – mammosphere forming efficiency, error bars ± 1 S.E.M., * p<0.05, ** p<0.01, *** p<0.001.

In order to further investigate CXCR4 signalling in malignant cells, CXCR4 was retrovirally over-expressed in MCF7 cells using human codon optimised CXCR4 cDNA. FACS was used to verify a 5.9 fold increase in CXCR4 compared with the empty vector control (83.3% vs. 14.2%, Figure 5A).

Over-expression of CXCR4 significantly increased (p<0.001) the mammosphere forming potential of MCF7 cells compared with empty vector control (Figure 5B), correlating with the data from the CXCR4 sort (Figure 4B). Second generation mammosphere culture demonstrated that over-expressing CXCR4 significantly increases self-renewal of MCF7 cells (p<0.001; Figure 5B). SDF-1 treatment alone or in combination with AMD3100 significantly increased the first generation MFE of the empty vector control cells (both p<0.01; Figure 5C), as previously seen with parental MCF7 cells. Addition of AMD3100 significantly reduced the MFE of the CXCR4 over-expressing cells (p<0.001) with combination treatment of AMD3100 and SDF-1 also showing a significant reduction (p<0.05).

The effects of CXCR4 over-expression on self-renewal were then investigated using the primary mammospheres formed after treatment with AMD3100 and SDF-1 (Figure 5D). Second generation mammosphere forming capacities of the empty vector control cells showed no difference across all treatment groups. In the CXCR4 over-expressing cells, the second generation mammosphere forming efficiency was significantly reduced in both AMD3100 treatment groups (both p<0.001). SDF-1 treatment had no effect on the secondary MFE of the CXCR4 over-expressing cells.

## DISCUSSION

In this study we utilised early mammosphere culture to harvest the anoikis-resistant population of immortalised normal and malignant breast cell lines. The anoikis-resistant fraction of the malignant cell line MCF7 has previously been demonstrated to be enriched for cancer stem cells [10]. We confirmed this previous finding by showing that anoikis-resistant cells can enrich for stem cells in the normal breast cell lines MCF10a and 226L and the malignant breast cell lines T47D and SKBR3. Normal stem cell enrichment was demonstrated using the clonogenic mammosphere and Matrigel assays *in vitro*. For the malignant cells, the mammosphere culture was used *in vitro* to confirm stem cell enrichment and limiting dilution assays *in vivo* to demonstrate increased tumour formation of the anoikis-resistant cells.

A custom gene microarray highlighted several genes that were differentially expressed between the stem cell-enriched populations and unsorted monolayer cells across all the cell lines (TRIM16, FOS, HES4 and ID1). Of particular interest was the increase in gene expression of CXCR4 in both the normal and malignant stem cell enriched fractions. Signalling of this chemokine receptor, via its ligand SDF-1, has been linked with migration in normal development and metastasis in many types of cancer [19, 29, 30]. More recently, high CXCR4 expression has been demonstrated in prostate, lung and pancreatic cancer stem cells, but the full extent of its role in cancer has not been elucidated [23-25]. In breast cancer, high CXCR4 expression is found in aggressive tumours, correlating with poor prognosis and a decrease in disease-free survival [31-33]. As well as a mediator of metastasis, CXCR4 signalling has been found to contribute to breast tumour growth at the primary site; however its function in stem cell activity, both normal and malignant, has not yet been investigated [19, 21].

Our data demonstrated that stimulation of the CXCR4 pathway in the normal breast cell lines by SDF-1 decreased mammosphere formation but had no effect on normal stem cell self-renewal. Inhibition of the CXCR4 pathway in the ER+ malignant cell line T47D, resulted in a significant increase in stem cell activity but a reduction in stem cell self-renewal. However, stimulation of the CXCR4 pathway in human primary fluid samples from metastatic breast cancer patients increased both stem cell activity and self-renewal. MCF7 cells either sorted for CXCR4 expression or transfected to over-express CXCR4 demonstrated greater mammosphere forming potential compared with controls suggesting an increase in stem cell activity. CXCR4 over-expression or stimulation of CXCR4 signalling conferred an increase in the self-renewal capacity of malignant stem cells.

This study is the first to explore the specific role of CXCR4 in normal and malignant breast stem cells. Our data is consistent with a model in which CXCR4 signalling produces contrasting effects in normal and malignant breast stem cells (Figure 5E). This could be due to the activation of different downstream targets. However, the effects on stem cell activity require further investigation and cannot be determined from the mammosphere assay alone. For example, the increase in stem cell activity observed in response to CXCR4 stimulation in malignant stem cells could result from an increase in proliferation, a release from senescence or an increase in anoikis resistance. Additional experimentation will be required to identify which of these functions is affected by CXCR4 signalling both *in vitro* and *in vivo*. The differences in the response of cells derived from human breast cancer and the malignant cells lines to AMD3100 and SDF-1 could be due to high levels of endogenous SDF-1 already present in the ascites fluid from which patient-derived samples were obtained (Table 1). Recent studies have demonstrated that CXCR7 can also heterodimerise with CXCR4, enhancing signalling via SDF-1 [34], adding complexity to the signalling pathway. The role of CXCR7 in cancer progression cannot be ignored, but distinguishing between the effects of CXCR4 and CXCR7 signalling will be difficult.

Previous studies in pancreatic and prostate cancer stem cells also showed that stimulation of CXCR4 signalling increased the cancer stem cell fraction, [24, 25] while inhibition reduced stem cell activity (measured by the ability to form metastases). Efforts to investigated the engraftment potential of CXCR4 sorted (by FACS) human cord blood and foetal cells have shown that CXCR4 negative cells engraft equally well as CXCR4 positive cells [35, 36]. Kollet *et al.* investigated this further and found that blocking CXCR4 signalling in the CXCR4 negative fraction inhibited engraftment. However, sorting for CXCR4 also reduced the engraftment of CXCR4 positive cells compared with unsorted cells. Interestingly, they also demonstrated that engraftment and migration of both sorted CXCR4 populations could be enhanced through the addition of an assortment of cytokines (including IL-6 and G-CSF). Overall, they propose that cells have intracellular stores of CXCR4 which can be released rapidly in response to cytokines and that sorting for CXCR4 using monoclonal antibodies which bind to same domains as SDF-1 can interfere with CXCR4 signalling. They suggest that cells should be sorted for CXCR4 expression using alternate methods such as migration assays.

A limitation with our predicted model is that it does not include the interaction of SDF-1 and its inhibitor with other receptors such as CXCR7 or the surrounding stroma which is responsible for the majority of SDF-1 secretion. The scavenging role of CXCR7 on SDF-1 may moderate CXCR4 signalling with high levels of expression of CXCR7 observed on many tumour cell lines and human primary tumour samples [37-39]. CXCR7 signalling has been demonstrated to increase cell proliferation and cellular adhesion suggesting that it may also function as a typical GPCR as well as a scavenging receptor, mediating the signal transduction process [37, 40, 41]. Whether these functional responses of CXCR7 observed are only via interaction with CXCR4 or are evidence of independent signalling are yet to be determined and requires further investigation.

## CONCLUSION

CXCR4 is expressed in a broad range of normal and malignant tissues and demonstrates diverse functions. This can be explained by changes in the activation of different downstream signalling components dependent on cell type and location. Our data establish for the first time that CXCR4 signalling has contrasting effects on normal and malignant breast stem cell activity. CXCR4 influences self-renewal of malignant stem cells and is highly expressed in stem cell populations in patient tumours, which may account for its role in tumour progression and metastasis. The differing role of CXCR4 signalling on normal and malignant breast stem cells suggests potential in the use of CXCR4-targeted therapy alongside current standards of care.

## MATERIALS AND METHODS

### Patient-derived breast cancer samples

Three ascites samples were collected from patients with metastatic breast cancer following fully informed consent (Local research ethics committee number 05/Q1402/25). All three samples were from patients with luminal (ER+/PR+) tumours, two of which were grade 3 (one HER2+, one HER2-) and had received chemo- and endocrine-therapy.

### Mice

All *in vivo* experiments utilised female athymic Nu/Nu (nude) mice obtained from Harlan laboratory. For experiments involving the estrogen receptor positive cell line T47D, estrogen pellets (17 β-oestradiol 90-day slow release pellets; Innovative Research of America) were implanted into mice 7 days before injection of the cells. Mice were monitored daily and weighed every 3-4 days.

### Cell lines and cell culture

MCF10a, MCF7 and T47D cell lines were purchased from ATCC (American Type Culture Collection). 226L cells were derived by infecting human breast epithelial cells with amphotropic retrovirus transducing a temperature-sensitive mutant of SV40 large T-antigen using the catalytic subunit of telomerase [42, 43] and were a gift from Professor Mike O'Hare (Ludwig Institute, London). They were cultured in Dulbecco's Modified Eagle's Medium (DMEM)/F12 (1:1 - Gibco) supplemented with 10% foetal bovine serum (FBS), 20ng/ml cholera toxin (Sigma), 20ng/ml human epidermal growth factor (hEGF), 5μg/ml insulin and 1μg/ml hydrocortisone. SKBR3 cells were a gift from Dr Valarie Speirs (Leeds University, UK) and cultured in McCoy's 5A (Gibco) with 10% FBS. HEK293-T fibroblast cells (derived from human kidney, ATCC) were used for production of virus and were a gift from Dr Akira Orimo (The University of Manchester, UK). MCF10a cells were cultured in DMEM/F12 (1:1) supplemented with 5% horse serum, 20ng/ml hEGF, 100ng/ml cholera toxin, 500ng/ml hydrocortisone and 10μg/ml insulin. MCF7, T47D and HEK293-T cells were grown in DMEM (Sigma), 10% FBS and 2mM L-glutamine. Levels of SDF-1 secreted into the media during 3 days of culture of cell lines were assayed for SDF-1 levels using a human CXCL12/SDF-1α Quantikine® ELISA, performed following the manufacturers protocol. All cells were incubated at 37°C and 5% humidified CO_2_ and were used in experiments when ~80% confluent. Cell lines were tested regularly for mycoplasma by PCR and discarded if positive.

### Primary cell extraction and culture

Cells from ascites fluid were collected by centrifugation at 1000g for 10 minutes at 4°C. The supernatant was collected and assayed for levels of SDF-1. The pellet was resuspended in cold PBS and red blood cells removed using Lymphoprep (Axis Shield). Magnetic-activated cell sorting (MACS) was used to remove lymphocytes and purify the remaining cells.

### Mammosphere culture

First and second generation mammosphere culture was performed following the previously published protocol [44]. All cell lines were plated out at a density of 500 viable cells/cm^2^ into mammosphere culture, except for MCF7 cells, which were plated out at a density of 300 viable cells/cm^2^. Mammospheres from primary samples were counted after 7 days in culture. AMD3100 (Sigma) and SDF-1 (Miltenyi) were added at time of plating into mammosphere culture at concentrations listed in each relevant figure. Media was not changed throughout the duration of the mammosphere culture.

### Harvesting anoikis-resistant cells

Cells from each epithelial cell line were plated out according to the first generation mammosphere culture protocol. After 8 and 12 hours the media containing the anoikis-resistant cells was harvested and centrifuged at 580g for 2 minutes. The supernatant was discarded, the cell pellet washed twice in cold PBS and the dead cells removed using a dead cell removal kit in conjunction with MACS® sorting columns (Miltenyi Biotec) following the manufacturers protocol (supplementary Figure 1).

### Matrigel culture

Twelve hour anoikis-resistant 226L or MCF10a cells were collected as described above. Single cell suspensions of monolayer 226L or MCF10a cells were also harvested. 50μl of Matrigel (BD Biosciences) was added to each well of a pre-chilled 8 well chamber slide and incubated at 37°C for 15 minutes to allow the basement membrane to solidify. Cells were resuspended in 2% Matrigel diluted in the respective monolayer media for each cell line, and 3000 cells plated out into each well in triplicate. Matrigel cultures were incubated at 37°C and 5% humidified CO_2_. Structures were scored after 21 days using a microscope fitted with a graticule.

### Limiting dilution assays in vivo

Twelve hour anoikis-resistant cells were collected from SKBR3 and T47D cell lines and the dead cells removed. Viable cells were resuspended in 50% Matrigel (v/v with PBS) and injected subcutaneously into each flank of nude mice assessing tumour growth every 3-4 days, measured using callipers. Experiments were ended when tumour growth plateaued or total tumour volume exceeded 1.3cm^3^. Tumour volumes were calculated as (length × length × width) divided by 2.

### Measuring CXCR4 surface expression

To analyse CXCR4 cell surface expression, fluorescent-activated cell sorting (FACS) was utilised. Single cell suspensions were centrifuged (580g for 2 minutes) and resuspended in cold PBS at ≤ 1 × 10^7^ cells/ml containing UV live/dead dye (Invitrogen). CXCR4 PE-Cy™5 (BD Biosciences) was added and the cells were incubated at 4°C for 15 minutes. The cells were washed with cold PBS, centrifuged at 1800g for 2 minutes and resuspended in cold PBS ready for analysis. Fluorescence was measured using the LSR II (BD Biosciences) and analysed using FlowJo (version 7.6.5).

### Sorting for CXCR4 expression

Staining of the cells for sorting followed the same protocol for analysis of surface CXCR4 expression except that the cells were resuspended in running buffer prior to sorting and a violet live/dead dye (Invitrogen) was used due to laser restrictions. Cells were sorted using the FACS Aria (Becton Dickinson) at 16 pounds per square inch (PSI) pressure and Hank's buffered saline solution (HBSS) as sheath fluid. For each sort, a sample with only viable dye was run as a pressure control.

### RNA extraction

RNA was collected from monolayer samples and anoikis-resistant cells harvested after 8 and 12 hours in mammosphere culture. RNA was extracted using the RNeasy® Plus Mini following the manufacturer's protocol, using a QIAshredder spin column (Qiagen) to homogenise the cell lysates. In addition to the gDNA eliminator spin column provided in the RNeasy kit, an on-column DNase digestion was performed to ensure maximum removal of DNA. The concentration of RNA was measured using a GeneQuant machine (Amersham Biosciences).

### Quantitative real time (RT)-PCR

Quantitative RT-PCR was performed to examine the levels of CXCR4 expression. For primer sequences see supplementary table 1. Samples were set up in triplicate, prepared using the EpMotion 5070 pipetting robot and analysed using the 7900 PCR machine (Applied Biosystems). Data was analysed using the relative quantification method normalising sample CT values with 3 housekeeper genes and gene expression was calculated as 2^−ΔΔCt^

### Agilent gene custom microarray

RNA integrity was assessed by microanalysis (Agilent Bioanalyser). RNA was amplified using the WT-Ovation™ Pico RNA Amplification System (NuGEN) following the manufacturers protocol which employs SPI™ amplification. The cDNA generated was fluorescently labelled using the FL-Ovation™ cDNA Fluorescent Module kit (NuGEN) with a single tag (Cy™3) (following manufacturers protocol). Custom microarray chips were purchased from Agilent using a web-based application to design the microarrays to our specifications. It was designed by selecting 7198 genes of interest and 4543 housekeeper genes. The genes of interest were selected on the basis of their implication in: stem cell maintenance, stem cell, proliferation and differentiation and cancer treatment resistance. The fluorescently tagged cDNA was loaded onto microarray slides and the Agilent microarray chips fastened carefully on top (8 chips to a slide). These were incubated at 65°C for 40 hours in a hybridisation oven to allow hybridisation to occur. The chips were scanned using an Agilent scanner.

### Gene expression analysis

Analysis of variance (ANOVA), further moderated by an empirical Bayes adjustment was used to analyse the Agilent gene expression data using SPSS (version 16.0). Heat maps were generated using GENE-E (version 1.0.397). Raw .cel files representing gene expression data of flow sorted cells from Creighton et al. [28] were downloaded from NCBI GEO (GSE7513), summarised with Ensembl alternative CDF [45] and normalised with RMA [46].

### CXCR4 over-expression using retro-virus

Packaging and envelope plasmids (pUMVC3-gag-pol and pCMV-VSVG ; Addgene plasmid 8449 and 8454) were expanded using Library Efficiency® DH5α™ Competent Cells (Invitrogen) following the standard transformation procedure on L-broth agar plates supplemented with ampicillin [47]. Human codon-optimised CXCR4 cDNA [48] was cloned into the pBABE retroviral vector (pBABE-puro; Addgene plasmid 1764).

Retro-virus particles were produced using the fibroblast cell line HEK293T. Transfection was performed after 24 hours using the FuGENE 6 kit following the manufacturer's protocol combining the packaging and envelope vectors with each construct. The media was changed 16 hours after transfection to standard HEK293T media. After 48 hours transfection, the media containing virus particles was collected and centrifuged at 900g for 10 minutes at 4°C to pellet any cells. The supernatant was passed through a 0.45μm filter to remove any remaining cells or debris. The virus was purified by layering onto a 20% sucrose cushion (v/v in PBS) and collected by ultracentrifugation at 33,735g for 3 hours at 4°C. MCF7 cells were infected with the virus 24 hours after plating by adding the virus to target cell media containing protamine sulphate (1:1000 - Sigma). Virus concentrations were determined by titration. Antibiotic selection of MCF7 infected cells was by puromycin (Invitrogen).

### Statistical analysis

Parametric tests were used to test for significant differences as all the data was normally distributed. Analysis of variance (ANOVA) with least significant difference (LSD) *post-hoc* comparisons was used when more than 2 groups were being assessed. Otherwise, unpaired t-tests were used for comparisons of 2 groups. Further statistical details about each experiment are stated in the figure legends. Each cell line was analysed separately unless stated. All statistical analysis was performed using SPSS software (version 16.0).

## SUPPLEMENTARY FIGURE AND TABLE





## References

[1] Smalley M, Ashworth A (2003). Stem cells and breast cancer: A field in transit. Nat Rev Cancer.

[2] Shackleton M, Vaillant F, Simpson KJ, Stingl J, Smyth GK, Asselin-Labat ML, Wu L, Lindeman GJ, Visvader JE (2006). Generation of a functional mammary gland from a single stem cell. Nature.

[3] Stingl J, Eirew P, Ricketson I, Shackleton M, Vaillant F, Choi D, Li HI, Eaves CJ (2006). Purification and unique properties of mammary epithelial stem cells. Nature.

[4] Van Keymeulen A, Rocha AS, Ousset M, Beck B, Bouvencourt G, Rock J, Sharma N, Dekoninck S, Blanpain C (2011). Distinct stem cells contribute to mammary gland development and maintenance. Nature.

[5] Al-Hajj M, Wicha MS, Benito-Hernandez A, Morrison SJ, Clarke MF (2003). Prospective identification of tumorigenic breast cancer cells. Proc Natl Acad Sci U S A.

[6] Li X, Lewis MT, Huang J, Gutierrez C, Osborne CK, Wu MF, Hilsenbeck SG, Pavlick A, Zhang X, Chamness GC, Wong H, Rosen J, Chang JC (2008). Intrinsic resistance of tumorigenic breast cancer cells to chemotherapy. J Natl Cancer Inst.

[7] Ponti D, Costa A, Zaffaroni N, Pratesi G, Petrangolini G, Coradini D, Pilotti S, Pierotti MA, Daidone MG (2005). Isolation and in vitro propagation of tumorigenic breast cancer cells with stem/progenitor cell properties. Cancer Res.

[8] Fillmore CM, Kuperwasser C (2008). Human breast cancer cell lines contain stem-like cells that self-renew, give rise to phenotypically diverse progeny and survive chemotherapy. Breast Cancer Res.

[9] Wright MH, Calcagno AM, Salcido CD, Carlson MD, Ambudkar SV, Varticovski L (2008). Brca1 breast tumors contain distinct CD44+/CD24- and CD133+ cells with cancer stem cell characteristics. Breast Cancer Res.

[10] Harrison H, Farnie G, Howell SJ, Rock RE, Stylianou S, Brennan KR, Bundred NJ, Clarke RB (2010). Regulation of breast cancer stem cell activity by signaling through the Notch4 receptor. Cancer research.

[11] Borgna S, Armellin M, di Gennaro A, Maestro R, Santarosa M (2012). Mesenchymal traits are selected along with stem features in breast cancer cells grown as mammospheres. Cell Cycle.

[12] Kohandel M (2012). Mesenchymal traits and cancer stem cells in mammospheres: chicken or egg?. Cell Cycle.

[13] Dontu G, Al-Hajj M, Abdallah WM, Clarke MF, Wicha MS (2003). Stem cells in normal breast development and breast cancer. Cell Prolif.

[14] Balkwill F (2004). The significance of cancer cell expression of the chemokine receptor CXCR4. Semin Cancer Biol.

[15] Zlotnik A (2006). Chemokines and cancer. Int J Cancer.

[16] Müller A, Homey B, Soto H, Ge N, Catron D, Buchanan ME, McClanahan T, Murphy E, Yuan W, Wagner SN, Barrera JL, Mohar A, Verástegui E, Zlotnik A (2001). Involvement of chemokine receptors in breast cancer metastasis. Nature.

[17] Chen Y, Stamatoyannopoulos G, Song CZ (2003). Down-regulation of CXCR4 by inducible small interfering RNA inhibits breast cancer cell invasion in vitro. Cancer Res.

[18] Liang Z, Wu T, Lou H, Yu X, Taichman RS, Lau SK, Nie S, Umbreit J, Shim H (2004). Inhibition of breast cancer metastasis by selective synthetic polypeptide against CXCR4. Cancer Res.

[19] Smith MC, Luker KE, Garbow JR, Prior JL, Jackson E, Piwnica-Worms D, Luker GD (2004). CXCR4 regulates growth of both primary and metastatic breast cancer. Cancer Res.

[20] Kang H, Watkins G, Parr C, Douglas-Jones A, Mansel RE, Jiang WG (2005). Stromal cell derived factor-1: its influence on invasiveness and migration of breast cancer cells in vitro, and its association with prognosis and survival in human breast cancer. Breast Cancer Res.

[21] Lapteva N, Yang AG, Sanders DE, Strube RW, Chen SY (2005). CXCR4 knockdown by small interfering RNA abrogates breast tumor growth in vivo. Cancer Gene Ther.

[22] Liang Z, Wu H, Reddy S, Zhu A, Wang S, Blevins D, Yoon Y, Zhang Y, Shim H (2007). Blockade of invasion and metastasis of breast cancer cells via targeting CXCR4 with an artificial microRNA. Biochem Biophys Res Commun.

[23] Bertolini G, Roz L, Perego P, Tortoreto M, Fontanella E, Gatti L, Pratesi G, Fabbri A, Andriani F, Tinelli S, Roz E, Caserini R, Lo Vullo S, Camerini T, Mariani L, Delia D (2009). Highly tumorigenic lung cancer CD133+ cells display stem-like features and are spared by cisplatin treatment. Proceedings of the National Academy of Sciences of the United States of America.

[24] Hermann PC, Huber SL, Herrler T, Aicher A, Ellwart JW, Guba M, Bruns CJ, Heeschen C (2007). Distinct populations of cancer stem cells determine tumor growth and metastatic activity in human pancreatic cancer. Cell Stem Cell.

[25] Miki J, Furusato B, Li H, Gu Y, Takahashi H, Egawa S, Sesterhenn IA, McLeod DG, Srivastava S, Rhim JS (2007). Identification of putative stem cell markers, CD133 and CXCR4, in hTERT-immortalized primary nonmalignant and malignant tumor-derived human prostate epithelial cell lines and in prostate cancer specimens. Cancer Res.

[26] Liu C, Levenstein M, Chen J, Tsifrina E, Yonescu R, Griffin C, Civin CI, Small D (1999). SZF1: a novel KRAB-zinc finger gene expressed in CD34+ stem/progenitor cells. Experimental hematology.

[27] Gemelli C, Montanari M, Tenedini E, Zanocco Marani T, Vignudelli T, Siena M, Zini R, Salati S, Tagliafico E, Manfredini R, Grande A, Ferrari S (2006). Virally mediated MafB transduction induces the monocyte commitment of human CD34+ hematopoietic stem/progenitor cells. Cell Death Differ.

[28] Creighton CJ, Li X, Landis M, Dixon JM, Neumeister VM, Sjolund A, Rimm DL, Wong H, Rodriguez A, Herschkowitz JI, Fan C, Zhang X, He X, Pavlick A, Gutierrez MC, Renshaw L (2009). Residual breast cancers after conventional therapy display mesenchymal as well as tumor-initiating features. Proc Natl Acad Sci U S A.

[29] Kijowski J, Baj-Krzyworzeka M, Majka M, Reca R, Marquez LA, Christofidou-Solomidou M, Janowska-Wieczorek A, Ratajczak MZ (2001). The SDF-1-CXCR4 axis stimulates VEGF secretion and activates integrins but does not affect proliferation and survival in lymphohematopoietic cells. Stem Cells.

[30] Phillips RJ, Burdick MD, Lutz M, Belperio JA, Keane MP, Strieter RM (2003). The stromal derived factor-1/CXCL12-CXC chemokine receptor 4 biological axis in non-small cell lung cancer metastases. Am J Respir Crit Care Med.

[31] Kato M, Kitayama J, Kazama S, Nagawa H (2003). Expression pattern of CXC chemokine receptor-4 is correlated with lymph node metastasis in human invasive ductal carcinoma. Breast Cancer Res.

[32] Schmid BC, Rudas M, Rezniczek GA, Leodolter S, Zeillinger R (2004). CXCR4 is expressed in ductal carcinoma in situ of the breast and in atypical ductal hyperplasia. Breast Cancer Res Treat.

[33] Salvucci O, Bouchard A, Baccarelli A, Deschenes J, Sauter G, Simon R, Bianchi R, Basik M (2006). The role of CXCR4 receptor expression in breast cancer: a large tissue microarray study. Breast Cancer Res Treat.

[34] Levoye A, Balabanian K, Baleux F, Bachelerie F, Lagane B (2009). CXCR7 heterodimerizes with CXCR4 and regulates CXCL12-mediated G protein signaling. Blood.

[35] Rosu-Myles M, Gallacher L, Murdoch B, Hess DA, Keeney M, Kelvin D, Dale L, Ferguson SS, Wu D, Fellows F, Bhatia M (2000). The human hematopoietic stem cell compartment is heterogeneous for CXCR4 expression. Proc Natl Acad Sci U S A.

[36] Kollet O, Petit I, Kahn J, Samira S, Dar A, Peled A, Deutsch V, Gunetti M, Piacibello W, Nagler A, Lapidot T (2002). Human CD34(+)CXCR4(−) sorted cells harbor intracellular CXCR4, which can be functionally expressed and provide NOD/SCID repopulation. Blood.

[37] Burns JM, Summers BC, Wang Y, Melikian A, Berahovich R, Miao Z, Penfold ME, Sunshine MJ, Littman DR, Kuo CJ, Wei K, McMaster BE, Wright K, Howard MC, Schall TJ (2006). A novel chemokine receptor for SDF-1 and I-TAC involved in cell survival, cell adhesion, and tumor development. J Exp Med.

[38] Goldmann T, Dromann D, Radtke J, Marwitz S, Lang DS, Schultz H, Vollmer E (2008). CXCR7 transcription in human non-small cell lung cancer and tumor-free lung tissues; possible regulation upon chemotherapy. Virchows Arch.

[39] Wang J, Shiozawa Y, Wang Y, Jung Y, Pienta KJ, Mehra R, Loberg R, Taichman RS (2008). The role of CXCR7/RDC1 as a chemokine receptor for CXCL12/SDF-1 in prostate cancer. J Biol Chem.

[40] Begley LA, MacDonald JW, Day ML, Macoska JA (2007). CXCL12 activates a robust transcriptional response in human prostate epithelial cells. J Biol Chem.

[41] Miao Z, Luker KE, Summers BC, Berahovich R, Bhojani MS, Rehemtulla A, Kleer CG, Essner JJ, Nasevicius A, Luker GD, Howard MC, Schall TJ (2007). CXCR7 (RDC1) promotes breast and lung tumor growth in vivo and is expressed on tumor-associated vasculature. Proc Natl Acad Sci U S A.

[42] Stamps AC, Davies SC, Burman J, O'Hare MJ (1994). Analysis of proviral integration in human mammary epithelial cell lines immortalized by retroviral infection with a temperature-sensitive SV40 T-antigen construct. Int J Cancer.

[43] O'Hare MJ, Bond J, Clarke C, Takeuchi Y, Atherton AJ, Berry C, Moody J, Silver AR, Davies DC, Alsop AE, Neville AM, Jat PS (2001). Conditional immortalization of freshly isolated human mammary fibroblasts and endothelial cells. Proc Natl Acad Sci U S A.

[44] Shaw FL, Harrison H, Spence K, Ablett MP, Simoes BM, Farnie G, Clarke RB (2012). A detailed mammosphere assay protocol for the quantification of breast stem cell activity. J Mammary Gland Biol Neoplasia.

[45] Dai M, Wang P, Boyd AD, Kostov G, Athey B, Jones EG, Bunney WE, Myers RM, Speed TP, Akil H, Watson SJ, Meng F (2005). Evolving gene/transcript definitions significantly alter the interpretation of GeneChip data. Nucleic Acids Res.

[46] Irizarry RA, Bolstad BM, Collin F, Cope LM, Hobbs B, Speed TP (2003). Summaries of Affymetrix GeneChip probe level data. Nucleic Acids Res.

[47] Stewart SA, Dykxhoorn DM, Palliser D, Mizuno H, Yu EY, An DS, Sabatini DM, Chen IS, Hahn WC, Sharp PA, Weinberg RA, Novina CD (2003). Lentivirus-delivered stable gene silencing by RNAi in primary cells. RNA.

[48] Babcock GJ, Farzan M, Sodroski J (2003). Ligand-independent dimerization of CXCR4, a principal HIV-1 coreceptor. J Biol Chem.

