# Supplementary material for: A differential role for CXCR4 in the regulation of normal versus malignant breast stem cell activity
Source: Oncotarget. 2013 Jul 30;5(3):599–612. doi: 10.18632/oncotarget.1169 (PMC3996659; doi:10.18632/oncotarget.1169)
Supplement: Supplementary file 1 [file oncotarget-05-0599-s001.pdf]

A differential role for CXCR4 in the regulation of normal versus malignant breast stem cell activity – Ablett et al

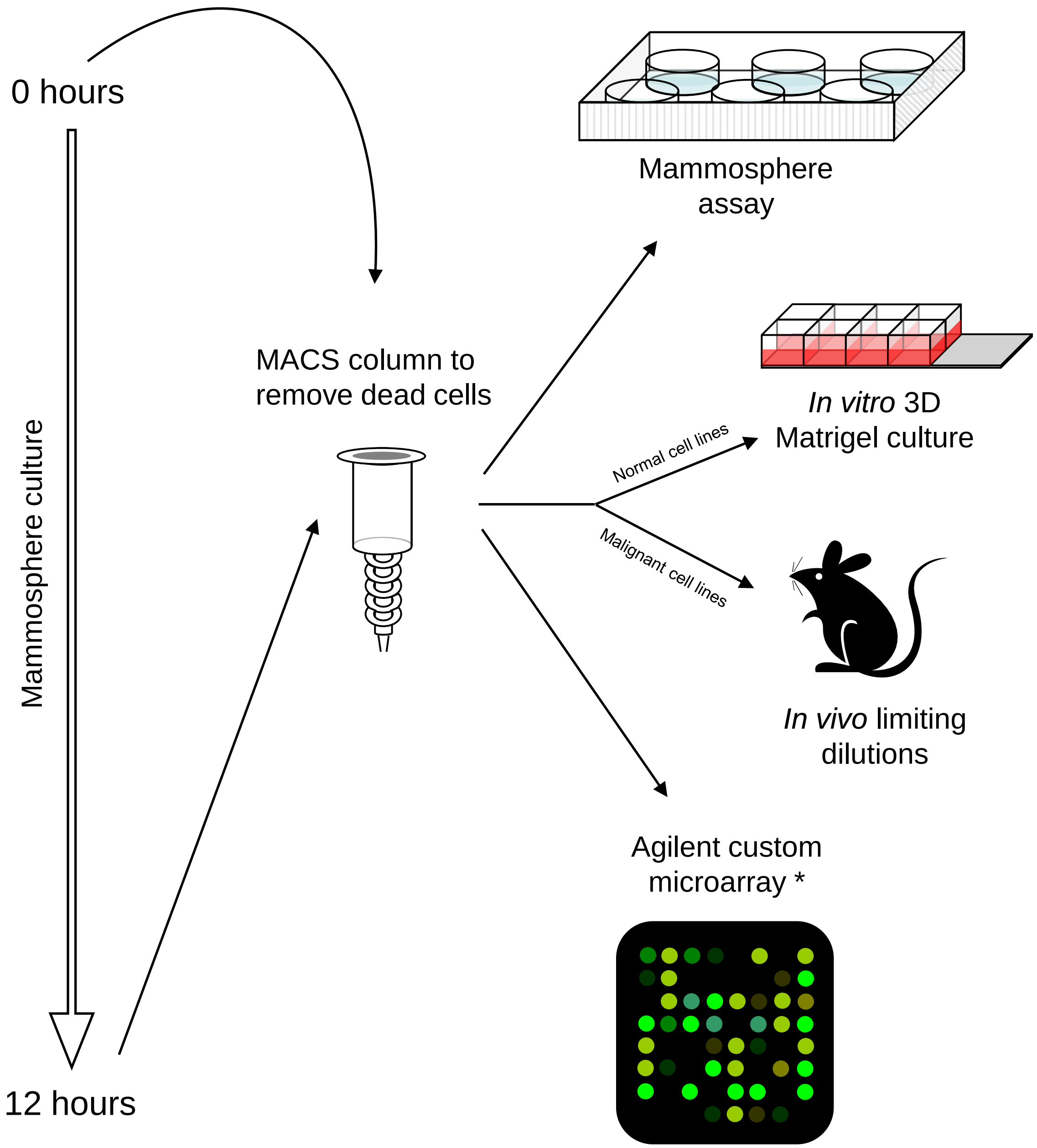

Supplementary Figure 1: Schematic representation of the method used to isolate anoikis-resistant (AR) cells from mammosphere culture and the assays utilised to validate stem cell enrichment. Cells harvested after 0 and 12 hours in mammosphere culture conditions were stained with a Live/Dead kit and passed through a MACS column to remove dead cells. The viable populations were then used in various assays to validate and quantify the degree of stem cell enrichment achieved from collecting AR cells. * Cells were collected after 0, 8 and 12 hour s in mammosphere culture for use in the Agilent microarray
