# Supplementary material for: A differential role for CXCR4 in the regulation of normal versus malignant breast stem cell activity
Source: Oncotarget. 2013 Jul 30;5(3):599–612. doi: 10.18632/oncotarget.1169 (PMC3996659; doi:10.18632/oncotarget.1169)
Supplement: Supplementary file 2 [file oncotarget-05-0599-s002.pdf]

A differential role for CXCR4 in the regulation of normal versus malignant breast stem cell activity – Ablett et al

Supplementary table 1: Sequences of the forward and reverse primers used for qRT-PCR.

Gene	Forward primer	Reverse primer
CXCR4	ATTGGGATCAGCATCGACTC	CAAACTCACACCCTTGCTTG
B2M	ATCTGAGCAGGTTGCTCCAC	GACCAAGATGTTGATGTTGGATAA
β-actin	CCAACCGCGAGAAGATGA	CCAGAGGCGTACAGGGATAG
GAPDH	AGCCACATCGCTCAGACAC	GCCCAATACGACCAAATCC
